# Data research on child abuse and neglect without informed consent? Balancing interests under Dutch law

**DOI:** 10.1007/s00431-015-2649-7

**Published:** 2015-10-21

**Authors:** Eva M. M. Hoytema van Konijnenburg, Arianne H. Teeuw, M. Corrette Ploem

**Affiliations:** Department of Pediatrics, Academic Medical Center, University of Amsterdam, Meibergdreef 9, 1105 AZ Amsterdam, The Netherlands; Department of Public Health, Academic Medical Center, University of Amsterdam, Meibergdreef 9, 1105 AZ Amsterdam, The Netherlands

**Keywords:** Child abuse, Research, Privacy/legislation & jurisprudence, Informed consent (by minors), Parental informed consent

## Abstract

According to the Declaration of Helsinki, participation of human subjects in medical research is only acceptable if subjects have given their consent. But in child abuse and neglect, many studies use a design in which subjects do not actively participate. Data in these studies are gathered from sources such as medical records or Child Protective Services. As long as such data are used anonymously, this does not interfere with individual privacy rights. However, some research is only possible when carried out with personally identifiable data, which could potentially be misused. In this paper, we discuss in which situations and under which conditions personal data of children may be used for a study without obtaining consent. In doing so, we make use of two recent studies, performed in our hospital, in which we encountered this issue. Both studies involved collecting personal data. After careful consideration, we decided not to ask informed consent; instead, we arranged for specific safeguards to protect the subject’s and their parents’ privacy as well as possible.

*Conclusion*: Altogether, we conclude that our approach fits within the Dutch legal framework and seems a reasonable solution in situations in which individual privacy rights are at odds with the public interest of child abuse and neglect research. We argue that, although, in principle, data research is only acceptable after informed consent is obtained, the law should allow that, under specific circumstances and safeguards, this requirement is put aside to make research in the field of child abuse and neglect possible.

**Table:** 

**What is known:** • *In principle, data research is only acceptable after informed consent is obtained.* • *In practice, this is not always feasible.*
**What is new:** • *Under specific circumstances and safeguards, the informed consent requirement can be put aside.*

## Introduction

According to the Declaration of Helsinki, participation of human subjects in medical research is only acceptable if subjects (or their legal representatives—e.g., parents) are fully informed of all aspects of the study and have given their consent [23]. However, in the field of child abuse and neglect, many studies use a design in which subjects do not actively participate. Such studies make use of available data about the children, their parents, and applied (health care) services and interventions to reduce child abuse and neglect. These data are gathered from sources such as medical records [10], records of Child Protective Services [22], and data provided by community services [18]. As long as such data are used anonymously, this does not interfere with individual privacy rights of the involved research subjects (which could be both the child and his or her parents). But in some situations, the use of anonymous data does not suffice, e.g., because the data needs deduplication or linking to data from other sources. In these situations, it is necessary to use personally identifiable (or personal) data, meaning that it is reasonably possible to trace an individual’s identity from the data (i.e., without a disproportionate use of means available to identify the person to whom the information relates). The use of such data could potentially cause problems or harm to the subjects or their parents when it is misused or ends up in wrong hands.

In research that is carried out in our hospital, we aim at linking data from different sources (medical records, records of Child Protective Services and community services, and self-reported data). This is only possible by using personal data. Whereas, we know that, in principle, all subjects should provide informed consent before their data is used, we experience that this is often not feasible, e.g., because the child and his or her parents are not traceable or—in case of large-scale database studies—it lacks means and time to approach each individual subject. Another problem is that in child abuse research, asking for informed consent may lead to a serious non-response bias [2, 11]. In this paper, we discuss in which situations and under which conditions a research project could be performed without obtaining informed consent of the research subjects. In doing so, we refer to two recent studies in our hospital in which we encountered these issues.

## Data research: two examples

### Example 1: evaluation of a protocol to identify child abuse and neglect

We conducted a study to evaluate a recently implemented hospital-based guidance protocol to improve identification of abused or neglected children in hospitals. This new protocol was based on a protocol developed in The Hague, The Netherlands in 2007 [8]. In the new protocol, all adults attending the emergency department because of medical problems due to intimate partner violence, substance abuse, or a suicide attempt were asked whether they care for children under 18 years of age. If so, children and their parents were referred to the outpatient pediatric department for an examination. After this visit, referrals to services could be arranged. If parents refused to cooperate after several reminders, children were reported to the Child Abuse Counseling and Reporting Centre (in Dutch: Advies en Meldpunt Kindermishandeling, AMK) [12]. If necessary, the AMK could decide to hand over serious cases to the Child Care and Protection Board (in Dutch: Raad voor de Kinderbescherming, RvdK), which is a division of the Ministry of Security and Justice [21]. To evaluate this protocol, we used several outcome measures. First, we used parent- and child-reported outcomes, for which we asked informed consent. However, based on previous studies and the opinion of other authors, we expected a low participation rate [7, 11, 20]. We were concerned that there would be a substantial non-response bias that would severely limit the external validity of the results. We expected that eligible subjects who were unwilling to participate in the study suffered from more problems than subjects who were willing to participate [4, 13–15]. In order to collect results from an unselected group, we wanted to include reports of hospital staff, AMK records, and RvdK records of all eligible subjects in the study (thus without asking for informed consent). Therefore, we would need to search AMK and RvdK records, and we needed to ask information from hospital staff. We could not use anonymous data, because we wanted to link data from different sources and, because the study was conducted in multiple hospitals, deduplicate. (Hospital staff could be unaware if subjects visited multiple hospitals during the study period).

### Example 2: validation of a screening test for child abuse and neglect

We conducted a study that aimed to validate two (already implemented) screening tests to identify child abuse and neglect at the hospital emergency department: a checklist called SPUTOVAMO and a complete physical inspection. All children visiting the emergency department underwent screening and were included in the study. As a reference standard, a multidisciplinary child protection team evaluated children with a positive screening result on either of the tests and assessed the presence of (a suspicion of) child abuse and neglect. This was all part of usual clinical care; data were used anonymously, and children and parents were not asked to provide informed consent. As a next step, however, we wanted to evaluate the proportion of false negative test results. These were children with negative results on both screening tests who were in reality victims of child abuse and neglect. For these children, and for children who had a positive screening test but were not evaluated by the multidisciplinary child protection team (missed diagnosis), we wanted to use AMK reports as a proxy reference standard for a child abuse diagnosis. Therefore, we needed to search the AMK database, which is not part of usual care and would require the use of personal identifiable data. After consideration, we did not want to ask informed consent for the AMK database search because, as with the previous study, we were concerned that parents who were maltreating their children would be less inclined to participate in the study. This would result in an overestimation of the sensitivity of the screening tests, with potentially dangerous consequences for future, maltreated children.

### Solution

After discussing our dilemma in a multidisciplinary team (including a hospital lawyer specialized in privacy matters, a dedicated hospital privacy officer, and a pediatrician specialized in child abuse and neglect), we used the following protocol in handling personal data: in both studies, only the main researcher (after signing a confidentiality agreement) searched for “hits” in the AMK and RvdK records. In the context of our approach, we could not inform subjects individually about our study, but in the majority of the participating hospitals, the general hospital leaflet contains a statement that, without objection, personal medical information can be used for research and patients will never be identifiable in any publications. There are instructions on how to object to this. If subjects would raise an objection against the use of their personal information in research, all information would be destroyed immediately. In the first study, only the main researcher collected all data from hospital staff. The researcher asked the staff to make a notification about participation in our study in the subjects’ records. During the study, all personal information was coded, and a key list was only kept by a trusted third party (a senior researcher, who was experienced in the field but not involved in the study). As soon as all data analyses were finished, all personal information was destroyed carefully.

## Legal framework for data research

### European level

When medical data research is involved, the main rules are provided by binding legislation (“hard law”) of the European Union (EU). We refer at a directive, adopted in 1995, on the protection of individuals with regard to the processing of personal data and on the free movement of such data (Directive 95/46/EC) [9]. By now, all EU-member states incorporated the directive’s provisions into their national laws. Although its framework on handling personal data of a particular sensitive nature, which medical data clearly are, is strict, it takes into account the importance of data processing for scientific research (in the medical field). It allows that the rights of individual “data subjects” (e.g., patients) are restricted when data processing for important research purposes is involved. The directive offers no more than a general legal framework. In this area, non-binding (“soft law”) rules and principles, developed by, e.g., the Council of Europe and the European Science Foundation, provide further guidance. The main principle embodied in these (binding and non-binding) documents is that medical data which are not anonymous, but (directly or indirectly) identifiable, may be collected and used for a research project if the data subject or his/her legal representative has given informed consent. However, when—despite reasonable efforts—it would be impracticable to seek the individual’s consent, the data may be gathered without the individual’s explicit consent, provided that certain conditions are met. One of these conditions is that the data subject knows about the possible use of his or her data for scientific research (right to be informed on a general level) and has not objected to this (right to “opt-out”) [16].

Presently, the European Union is in the middle of reforming the data protection framework. It is developing the so-called General Data Protection Regulation (GDPR), which is not only binding to the EU-member states (as the Directive) but also directly applicable in the member states, making implementation unnecessary. Because the draft regulation, once adopted, may leave less room for research with medical data than the present EU-framework, i.e., by providing stricter conditions for research in situations in which it would be impracticable to seek the individual’s consent, it is subject to strong debate inside and outside the medical research community [1, 17, 19]. It is important to note that, in June 2015, the Council of Ministers proposed a version of the GDPR that leaves substantially more room for research than the proposal of the European Parliament from May 2014; on the basis of the council’s version, broad consent seems to be possible, and further regulation of research without consent is left to the legislation of the member states [5]. However, the three EU-bodies (Council of Ministers, European Parliament, and European Commission) have to negotiate about the final text. This is not expected before the end of 2015.

### National level

In The Netherlands, in the context of medical data research, two laws are relevant: the Personal Data Protection Act (in Dutch: “Wet bescherming persoonsgegevens”) and the Medical Contract Act (in Dutch: Wet inzake de geneeskundige behandelingsovereenkomst), a section of the Dutch Civil Code. Here, we pay only attention to the latter because that is the one that provides specific rules for disclosing patient data for medical research. On the basis of Article 7:457 Civil Code, a physician or caregiver may disclose identifiable patient data to researchers when the patient authorized the disclosure. However, when obtaining consent appears to be impossible or would involve a disproportionate effort, that requirement may be dropped under the following conditions. First, the research project should serve a public interest; second, the research could not be carried out without the data concerned; third, the data subject is informed about the possible use of his/her data for research and has not explicitly opposed to this; and fourth, adequate security measures (technical and organizational) are taken (e.g., encoding the data).

We would like to note that, whereas this framework fits within the present EU-directive, it might come in conflict with the future EU-regulation.

## Discussion

In child abuse and neglect research, studies using linkable data of unselected samples are often necessary to yield unbiased results of sufficient quality. Since the results of these studies can have important consequences for the social, mental, and medical well-being of maltreated children and their families, we argue that, in very specific circumstances, it should be possible to perform such research without informed consent of the child and/or the parents involved. In this paper, we presented two examples: the first was evaluating a new protocol implemented to identify child abuse and neglect by assessing high risk parents, and the second was evaluating the diagnostic accuracy of two screening tools for maltreatment used in children at the emergency department. The most important reason not to ask informed consent was that we expected a severe non-response bias if we would only include families who were willing to participate. In both studies, the screening methods to identify child abuse and neglect that we wanted to investigate were already implemented in clinical care, although they had never been evaluated. We believe that good quality evaluation research is very important. However, in carrying out such research, the protection of privacy of research subjects may not, or at least as minimal as possible, be compromised. And for researchers, it is also very important that the performance of the study may not come into conflict with legal standards. According to Dutch law (Medical Contract Act; see before), consent can only be dropped if the research project serves a public interest, the project could not be carried out without the requested data, and the stored data are well secured by encoding them. And last but not least, the data subject is informed about the possible use of his/her data for research and has not explicitly opposed to this. With our approach, we were able to fulfill those conditions: the studies serve a public interest and could not be carried out without the data involved. As illustrated by the two examples as described before, we took several safeguards to ensure that the privacy of research subjects was protected as much as possible. First, we used an approach in which only the main researcher had access to personal data and no information was handed over to other parties (such as the AMK). In this way, the other parties could never use any information from the study in a way that could potentially negatively affect research subjects. If we would have asked the AMK to look for hits of our study participants in their databases, AMK staff could have used this information in their clinical assessments, which could have had consequences for the study subjects. The downside of having the researcher searching in databases of other parties is that the researcher could look up extra, non-relevant, information of study subjects or other persons, thus violating the privacy of these persons. Other than signing a confidentiality agreement, we could not be 100 % sure that this would not happen. Another safeguard was immediate coding of personal information, meaning that—by making use of a trusted third party (based in a different department of our institution)—all personal data were stored separately from the research data. By doing so, although not completely avoided, the risk that meaningful personal information would end up in wrong hands was minimal. We would like to note, however, that if financially feasible, it would be preferable, to use a completely independent trusted third party as a linkage center [3]. In this way, personal data are coded at the linkage center before any other information is retrieved from parties and send to researchers, and neither the other parties (such as the AMK) nor the researcher has access to any additional data [3]. Furthermore, research subjects and/or their representatives were informed about the possibility that their data could be used for medical research in general by a statement in the hospital leaflet or on the hospital website.

## Final remarks

In this paper, we discussed an approach to conduct research about child abuse and neglect without obtaining informed consent. This approach is consistent with the Dutch legal framework (therefore: the Dutch approach) and may be valuable for other countries. However, we argue that some improvements should be made.

First, we only partly fulfilled the “opt-out” condition for research subjects, because, although in theory, patients could read a statement about research based on patient records and instructions to object, most patients were probably not aware of this. We therefore recommend an improved opt-out procedure in the sense that it is better secured that patients and/or their legal representatives are really aware of the fact that their medical records could be used in research, and that they are always entitled to object to such use. This information should be available explicitly; hospitals could develop special leaflets for using medical data for research and put these posters or leaflets in waiting rooms, and the information could be put prominent on their websites. To prevent that patients opt out without even considering their participation, it should be clearly explained that the research is used to improve medical care, that personal information will never be distributed or published, and that patients will not experience any negative consequences. We think that a general leaflet, distributed to all patients visiting the hospital, informing and explaining them in clear and accessible language about the possibility of using their data for research purposes, would be the best way to go. In that way, patients will understand that data research is a normal part of healthcare.

Second, when researchers refrain from obtaining informed consent while they make use of personal data, we recommend a review of the research proposal by a medical ethics committee (MEC) or comparable body, especially in sensitive research areas as on child abuse and neglect. Although this is not a current requirement under Dutch law, it is recommended by non-binding documents such as the Council of Europe’s Recommendation No. R (97) 5 on the Protection of Medical Data (February 13, 1997) [6] (see Article 12.2 sub c). Although hospital or university medical ethics committees have no authority over external organizations that provide data, such as the AMK, they do have authority over the researchers working in their own institution. If their MEC does not approve the research protocol, the researcher is not allowed to proceed with the study. It would be even more careful if external organizations, such as the AMK, would only cooperate with extraction from its database if researchers hand over a MEC approval.

We would like to conclude that this approach seems a reasonable solution in situations in which individual privacy rights are at odds with the public interest of child abuse and neglect research. We argue that—although, in principle, medical research with human subjects is only acceptable after informed consent is obtained—European and national law should allow that under certain circumstances and safeguards the informed consent requirement is put aside to avoid that research in the field of child abuse will be (come) impossible.

## Abbreviations

AMK: Child Abuse Counseling and Reporting Centre (in Dutch: Advies en Meldpunt Kindermishandeling)
EU: European Union
RvdK: Child Care and Protection Board (in Dutch: Raad voor de Kinderbescherming)
